# To biofilm or not to biofilm

**DOI:** 10.7554/eLife.80891

**Published:** 2022-07-21

**Authors:** Shravan Pradeep, Paulo E Arratia

**Affiliations:** 1 Department of Earth and Environmental Sciences, University of Pennsylvania Philadelphia United States; 2 Department of Mechanical Engineering and Applied Mechanics, University of Pennsylvania Philadelphia United States

**Keywords:** biofilm, chemotaxis, motility, quorum sensing, dispersal, bacteria, None

## Abstract

A new model helps to predict under which conditions a species of bacteria will switch to a static lifestyle.

**Related research article** Moore-Ott JA, Chiu S, Amchin DB, Bhattacharjee T, Datta SS. 2022. A biophysical threshold for biofilm formation. *eLife* **11**:e76380. doi: 10.7554/eLife.76380.

A trip to the dentist is seldom fun, but it is often necessary to remove the sticky, slimy deposits (or biofilms) that adhere to our teeth and gums. These structures are formed by bacteria that have adopted a static lifestyle in the moist and warm environment of our mouths. In fact, biofilms are common in a range of natural, clinical, and industrial settings, where they can be dangerous for our health or contaminate equipments (Davey and O’Toole, 2000; Hall-Stoodley et al., 2004).

In general, bacteria can either exist in a mobile, ‘planktonic’ state where they freely disperse and explore their environment for nutrients, or stay statically as ‘biofilms’, a communal state where the cells share resources and are protected from harmful conditions (Adler, 1966). What triggers bacteria to transition from a mobile state to a biofilm lifestyle depends on how each species responds to certain environmental conditions. The factors include nutrient availability, production of certain chemical triggers as well as cellular parameters - such as bacterial concentration, proliferation rate, or diffusing behavior (Berg, 2018).

Overall, however, the switch to (immobile) biofilm formation is controlled by bacterial dispersion (which is dependent on nutrient levels), and it occurs when the concentration of bacterial molecules known as autoinducers goes above a certain threshold (Davies et al., 1998; Waters and Bassler, 2005). These signals, which are produced by bacteria, serve as a proxy for the level of other bacterial cells in the environment and trigger intracellular signals which impact the genes a cell expresses, and the lifestyle it will adopt. Once the biofilm is created, it is maintained by the autoinducer molecules produced by the immobilized bacteria (Figure 1).

**Figure 1.**
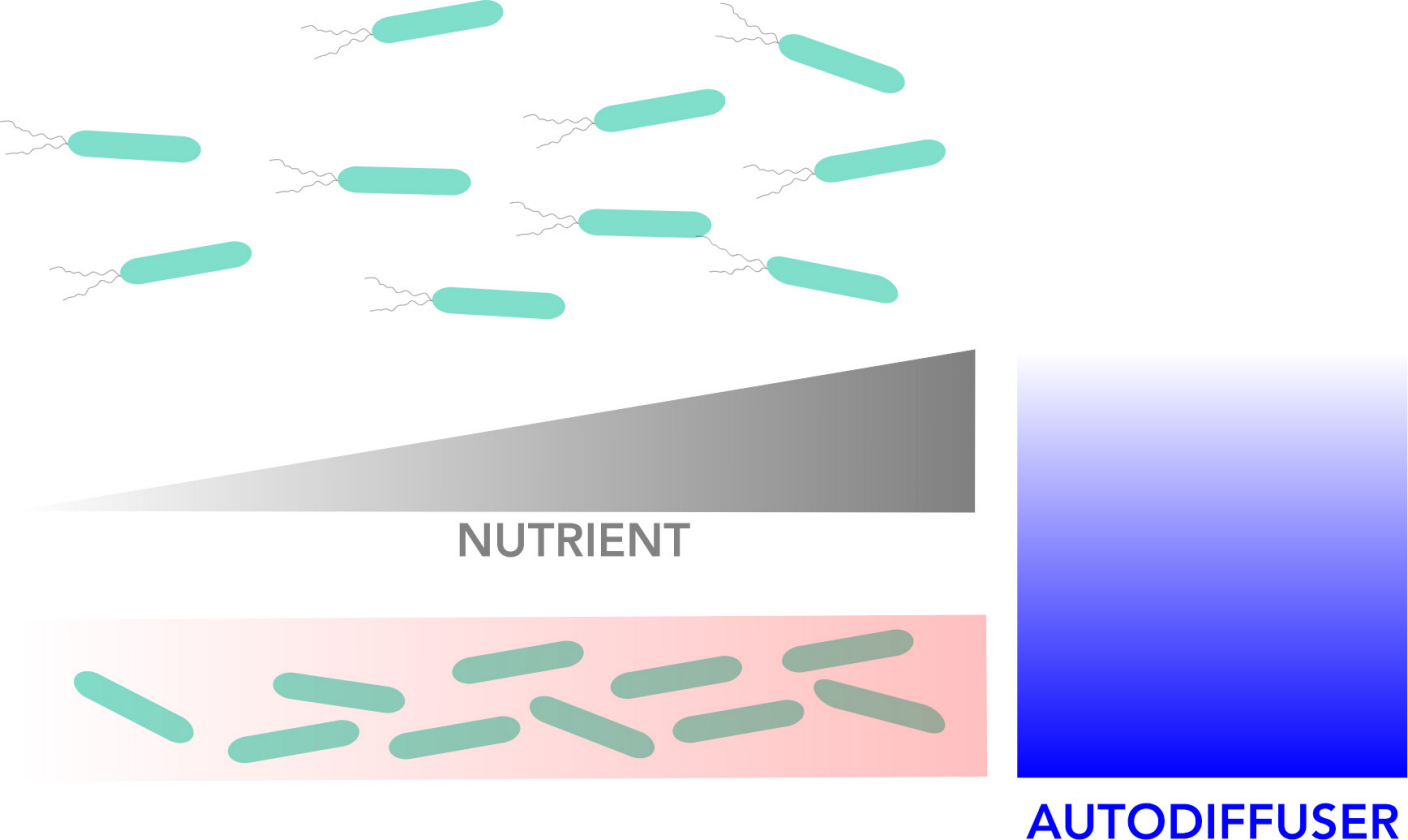
Visual representation of the model for biofilm formation. Bacteria can exist in two different states: a motile state in which they can disperse freely around their environment (top), and an immobile state in which they live together in static as a biofilm (bottom). The red gradient in the biofilm box indicates to which extent bacterial density is increasing in the biofilm from left to right alongside rising nutrient concentrations (grey gradient). The motile bacteria move towards increasing nutrient concentration to the right. The concentration of autodiffusers (molecules produced by bacteria which trigger biofilm formation; blue gradient), is highest close to the biofilm and decreases further away.

Yet, how biofilms emerge and the exact conditions that trigger their formation remain a topic of intense research. In general, motile and biofilm lifestyles are studied separately, making it difficult to predict with certainty whether a species of bacteria will form a biofilm under certain conditions. Now, in eLife, Sujit Datta and colleagues at Princeton University – including Jenna Moore-Ott as first author – report having developed a unified framework that can examine both states simultaneously (Moore-Ott et al., 2022).

The team developed a series of equations that describe the transition from planktonic state to biofilm under a range of parameters covering all possible conditions. The resulting model, which describes the behavior of the cells, is governed by two main factors: nutrient consumption and bacterial dispersion in the motile state. Both parameters focus on the competition between bacterial dispersion and the production of autoinducer molecules.

Based on the model, Moore-Ott et al. predict two conditions where the concentration of autoinducers remains under the threshold required for biofilm formation. In the first case, nutrients are consumed at such a high rate that the autoinducers are produced (by bacteria) in limited quantities; there is simply not enough autoinducer ‘production’ time. In the second case, bacteria diffuse and therefore disperse at increased levels (possibly because of environmental conditions), limiting the accumulation of the autoinducers in one location.

In addition, Moore-Ott et al. also pinpointed a third factor the ratio between the time it takes for nutrients to be consumed and for autoinducer to be produced, which affects how fast the biofilm forms and how large they become. For instance, a larger ratio between these two timescales results in the biofilm proliferating, while a smaller ratio slows down the formation of the biofilm. Overall, the combination of these three parameters – nutrient consumption, bacterial dispersion, and ratio of consumption to production time scale – determine which lifestyle a specific species adopts, and at what concentration.

While nutrient consumption and bacterial dispersion vary between different species of bacteria and across environments, they are quantifiable through experiments. This means that the model provides a unique general framework that can be used to predict which state a given bacterial species will adopt under specific circumstances.

Further work should aim to refine the model so it can become closer to real life conditions. For example, the framework assumes that biofilm formation and the production of autoinducers in a nutrient-dependent fashion are irreversible, two assumptions which can be relaxed for certain species of bacteria (Bridges and Bassler, 2019). In addition, more complex elements could be added to tailor the framework to a specific system, such as incorporating how the biofilm is spatially organized, inputting the role of secondary signaling molecules which fine-tune the impact of autoinducers, or acknowledging how individual cells may respond differently to signals (Bhattacharjee et al., 2022; Jenal et al., 2017; Nadezhdin et al., 2020). Nevertheless, this work represents an important step forward in our quantitative understanding of biofilm formation, which in turn will help us in both fighting and harnessing biofilms, which can be useful in wound healing, bioremediation, or functional materials production.
